# *Ex vivo* ovine liver model simulating respiratory motion and blood perfusion for validating image-guided HIFU systems

**DOI:** 10.1186/2050-5736-3-S1-P53

**Published:** 2015-06-30

**Authors:** Xu Xiao, Markus Domschke, Benjamin Cox, Helen McLeod, Ioannis Karakitsios, Andreas Melzer

**Affiliations:** 1 University of Dundee, Dundee, United Kingdom

## Background/introduction

The effect of moving organs and blood perfusion on image guided procedures such as focused ultrasound is challenging therefore we have developed *ex vivo* ovine liver phantoms to simulate respiratory motion and blood perfusion. The simulator was used to validate ultrasound image-guided HIFU treatment when the target tissue was moving.

## Methods

**Figure 1:**
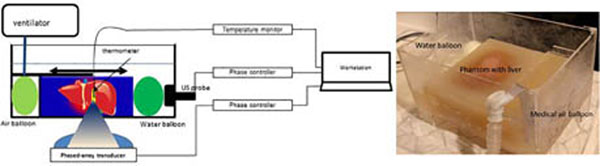
Illustration and photo of respiratory liver motion simulator, ovine liver is embedded into 3% agar-gelatine phantom

**Figure 2:**
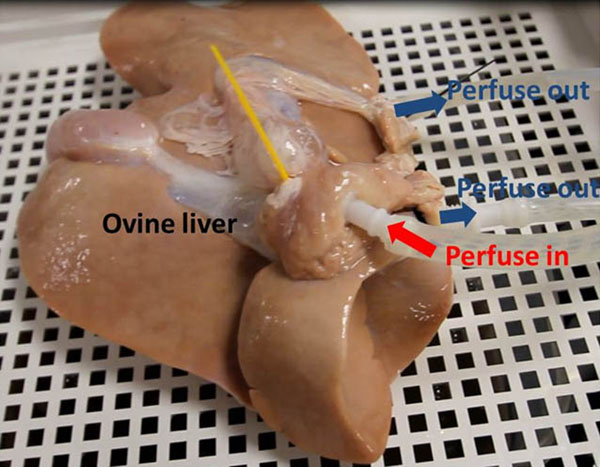
Setup of perfusion of ovine liver

The respiratory liver motion simulator consists of a physical ovine liver, agar-gelatine block surrounding the liver, a medical air balloon which is connected to a lung ventilator, and two water balloons which are used to move the phantom back to its original position. The whole setup was MR compatible, including the ventilator which is used to provide simulation of respiratory motion. The movement generated by the simulator was analysed via an MR compatible ultrasound system, and this real-time motion information was used to guide a dynamic high intensity focused ultrasound system (Exablate 2100 system, 0.55MHz) to steer its focus to follow this motion (Figure 1). The liver model is perfused via an extracorporeal circuit driven by a heart lung bypass machine (HL30 Maquet, Germany). Continuous non pulsatile input into the portal vein of 220 ml/min saline, with output free draining into the venous system, forming a perfusion circuit. (Figure 2). Digital Subtraction Angiography contrast images of the vasculature (OEC 9900, GE USA: Ultravist 370, Bayer HealthCare) to scanning with the MR and ultrasound doppler. The perfused vessel was then scanned in the 1.5 T Signa HDxGE (GE, USA) and ultrasound wireless scanner (Accuson SIEMENS, GERMANY).

## Results and conclusions

**Figure 3:**
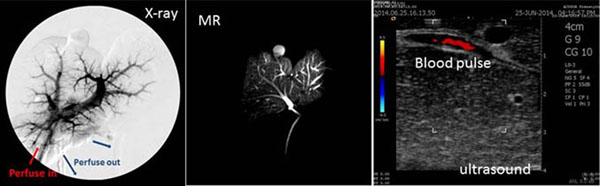
X-ray scan, and MR scan and ultrasound scan results of the ovine liver phantom

Mean displacement between expiration and inspiration was up to 30 mm along superior-inferior direction. The setup also allowed motion in left-right direction by reducing the width of the phantom block. After establishing the saline water perfusion, the vessels of the ovine liver provides a large contract from the background in x-ray and MR scan (FRFSE-XL, TE=8.7, TR=400). And a clear pulse was observed in the ultrasound colour Doppler (Figure 3). The ovine liver movement generated by the respiratory simulator is comparable to that of a human liver *in vivo*. This model could be used to test the influence of motion on image guided focused ultrasound therapy. The successful re-creation of physiological flow in the peripheral arteries of ovine liver has provided a model for testing the influence of blood flow rate on the image guided focused ultrasound sonication. Therefore the phantom provides a low-cost option for validating ultrasound and MR image-guided focused ultrasound treatment.

